# Surveillance and Genomics of Toxigenic *Vibrio cholerae* O1 From Fish, Phytoplankton and Water in Lake Victoria, Tanzania

**DOI:** 10.3389/fmicb.2019.00901

**Published:** 2019-04-30

**Authors:** Yaovi M. Gildas Hounmanou, Pimlapas Leekitcharoenphon, Rene S. Hendriksen, Tamegnon V. Dougnon, Robinson H. Mdegela, John E. Olsen, Anders Dalsgaard

**Affiliations:** ^1^Department of Veterinary and Animal Sciences, Faculty of Health and Medical Sciences, University of Copenhagen, Copenhagen, Denmark; ^2^National Food Institute, WHO Collaborating Center for Antimicrobial Resistance in Food Borne Pathogens and Genomics and European Union Reference Laboratory for Antimicrobial Resistance, Technical University of Denmark, Kongens Lyngby, Denmark; ^3^Research Unit in Applied Microbiology and Pharmacology of Natural Substances, Laboratory of Research in Applied Biology, Polytechnic School of Abomey-Calavi, University of Abomey-Calavi, Cotonou, Benin; ^4^Department of Veterinary Medicine and Public Health, College of Veterinary Medicine and Biomedical Sciences, Sokoine University of Agriculture, Morogoro, Tanzania

**Keywords:** *Vibrio cholerae*, genomics, aquatic reservoirs, African Great Lakes, microbial ecology

## Abstract

The occurrence of toxigenic *Vibrio cholerae* O1 during a non- outbreak period in Lake Victoria was studied and genetic characteristics for environmental persistence and relatedness to pandemic strains were assessed. We analyzed 360 samples of carps, phytoplankton and water collected in 2017 during dry and rainy seasons in the Tanzanian basin of Lake Victoria. Samples were tested using PCR (*omp*W and *ctx*A) with DNA extracted from bacterial isolates and samples enriched in alkaline peptone water. Isolates were screened with polyvalent antiserum O1 followed by antimicrobial susceptibility testing. Whole genome sequencing and bioinformatics tools were employed to investigate the genomic characteristics of the isolates. More *V. cholerae* positive samples were recovered by PCR when DNA was obtained from enriched samples than from isolates (69.0% vs. 21.3%, *p* < 0.05), irrespectively of season. We identified ten *V. cholerae* O1 among 22 *ctx*A-positive isolates. Further studies are needed to serotype the remaining *ctx*A-positive non-O1 strains. Sequenced strains belonged to El Tor atypical biotype of *V. cholerae* O1 of MLST ST69 harboring the seventh pandemic gene. Major virulence genes, *ctxA, ctxB, zot, ace, tcpA, hlyA, rtxA, ompU, toxR, T6SS, alsD, makA* and pathogenicity islands VPI-1, VPI-2, VSP-1, and VSP-2 were found in all strains. The strains contained *Vibrio* polysaccharide biosynthesis enzymes, the *mshA* gene and two-component response regulator proteins involved in stress response and autoinducers for quorum sensing and biofilm formation. They carried the SXT integrative conjugative element with phenotypic and genotypic resistance to aminoglycoside, sulfamethoxazole, trimethoprim, phenicol, and quinolones. Strains contained a multidrug efflux pump component and were resistant to toxic compounds with copper homeostasis and cobalt-zinc-cadmium resistance proteins. The environmental strains belonged to the third wave of the seventh pandemic and most are genetically closely related to recent outbreak strains from Tanzania, Kenya, and Uganda with as low as three SNPs difference. Some strains have persisted longer in the environment and were more related to older outbreak strains in the region. *V. cholerae* O1 of outbreak potential seem to persist in Lake Victoria through interactions with fish and phytoplankton supported by the optimum water parameters and intrinsic genetic features enhancing survival in the aquatic environment.

## Introduction

*Vibrio cholerae*, the causative agent of cholera, is one of the oldest pathogens known to cause disease in humans, yet there is still much to be learned regarding its transmission and evolution. Although the majority of the worldś cholera cases occur in Africa, there has been limited research on the occurrence of toxigenic *V. cholerae* O1 in aquatic environments in countries like Tanzania located around the African Great Lakes (Dalusi et al., 2015a; Hounmanou et al., 2016; Bwire et al., 2018a). Knowledge on occurrence and transmission in and around the lakes are important when establishing preventive and control measures of epidemic cholera, which often affects countries situated around the lakes. Moreover, *V. cholerae* is ubiquitous in aquatic environments and phytoplankton and fish have been reported as potential reservoirs, e.g., in Tanzania (Haque et al., 2012; Rabia et al., 2017; Hossain et al., 2018a; Nyambuli et al., 2018). The tropical waters of Lake Victoria and Lake Tanganyika are likely natural habitats for the El Tor variant of *V. cholerae* O1 (Echenberg, 2011).

The incidence of cholera in countries around the Great Lakes varies by season, rainfall, plankton blooms, and level of fishing activities (Nkoko et al., 2011). *V. cholerae* O1 resistant to common antimicrobials have been isolated in fish and water from the Kenyan side of Lake Victoria (Onyuka et al., 2011). A study in Lake Tanganyika indicated a strong correlation between planktonic blooms, fish abundance and cholera (Plisnier et al., 2015). A recent study in Uganda has also identified *V. cholerae* O1 in Lake Victoria although the strains were non-toxigenic (Bwire et al., 2018a).

Tanzania has consistently been affected by cholera since 1974 and outbreaks continue to re-occur in various towns, especially in the coastal and Great Lakes regions (Urassa et al., 2009; Reyburn et al., 2011). Since the 7th pandemic reached the country in 1974, cholera has affected more than 230,596 people causing 17,714 deaths, most of which lived in lake zones (Mengel et al., 2014). Despite suggestions of genetic similarity and clonality of environmental and clinical *V. cholerae* strains (Dalusi et al., 2015b), recent studies have revealed different clonal complexes based on MLVA typing among strains implicated in cholera outbreaks in Tanzania (Kachwamba et al., 2017). Analyses of cholera outbreak strains from Uganda and Mozambique however showed a high level of similarity among strains suggesting a clonal transmission in East African countries (Garrine et al., 2017; Bwire et al., 2018b) probably facilitated by the lakes they share. Thus, there is a need to determine the relatedness of environmental and clinical isolates of *V. cholerae* and the role of the aquatic lake environments as reservoirs for the pathogen.

Furthermore, *V. cholerae* has evolved through the emergence of multidrug resistant strains with the acquisition of the SXT integrative conjugative elements (Hendriksen et al., 2011; Spagnoletti et al., 2014; Kaas et al., 2016) and transfer of the cholera toxin gene through phages (Waldor and Mekalanos, 1996). The emergence of the El Tor variant biotype carrying the *ctx*B1 gene of the Classical biotype and *V. cholerae* O1 carrying the *ctx*B7 gene of the Haitian strain is another example of evolution of *V. cholerae* and stress the importance of a continuous monitoring of the genetic characteristics of environmental *V. cholerae* (Ghosh-Banerjee et al., 2010; Kim et al., 2014).

The aim of this study was to investigate the occurrence of toxigenic *V*. *cholerae* O1 during a non-cholera outbreak period in Lake Victoria and determine the genetic characteristics that support environmental persistence and genetic relatedness to pandemic strains. Whole genome sequencing (WGS) and bioinformatics analysis coupled with environmental surveillance data were applied to determine the occurrence and outbreak potential of *V. cholerae* O1 in Lake Victoria. The data generated add to our understanding on aquatic reservoirs of toxigenic *V. cholerae* O1 in Lake Victoria.

## Materials and Methods

### Sampling Area and Sample Collection

**FIGURE 1:**
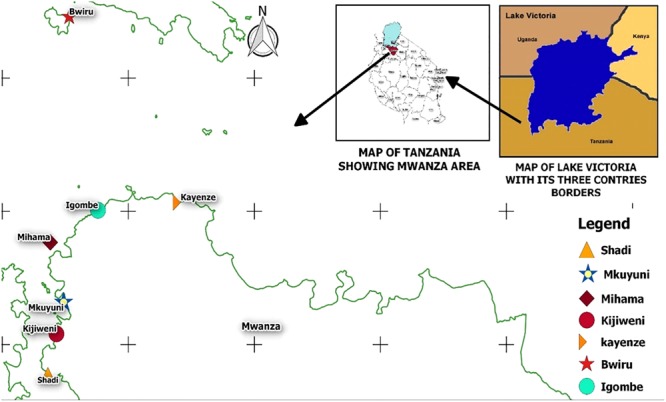
Sampling points in Lake Victoria, Mwanza, Tanzania.

During the dry season of June to September 2017 and the rainy season of October to December 2017, 360 samples of lake water, carps (*Rastrineobola argentea*) and phytoplankton were collected from seven landing sites in the Mwanza Gulf of the Tanzanian basin of Lake Victoria (Figure 1). These included 120 water samples (60 offshore and 60 collected near the lakeshore), 120 carp samples and 120 phytoplankton samples (vertical and horizontal sampling as described below). Samples were collected during both seasons (180 samples in the dry season and 180 samples in the rainy season). Sampling sites were Shadi, Kijiweni and Mkuyuni in Mwanza South and Igombe, Mihama, Bwiru and Kayenze in Mwanza North. Mwanza is located in the Lake Victoria basin between the latitude -2° 31′ 0.01″ S and the longitude 32° 53′ 60.00″ E (Figure 1). Samples of fresh carp (about 200 g per sample, consisting of about 200 individual fish) were purchased from local fishermen immediately after landing at the beach and placed in sterile labeled plastic bags which were transported to the National Fish Quality Control Laboratory (NFQCL) in Mwanza in an insulated box with cooling elements and processed within 4 h of collection.

Surface water samples were collected both onshore and offshore (at least 200 m away from the nearest shore) and were collected in 250 ml sterile glass bottles and transported as shown above for carp samples.

Approximately 100 ml of phytoplankton samples were collected by boat with a phytoplankton towing net of 13-μm mesh size. To maximize chances of recovering *V. cholerae* from the Lake, phytoplankton samples were obtained from the water surface (horizontal sampling) and from beneath to top (vertical sampling) in order not to miss species/taxa that do not float on the surface water which could harbor *V. cholerae* of interest. For horizontal samples, the net was set right below the surface of the water after which the boat moved about 50 m. The net was then raised and the phytoplankton sample collected. Vertical samples were collected at a minimum depth of 5 m (up to 10 m depending on the depth offshore) where the net was submerged with a lead to collect the phytoplankton sample by a movement from bottom to top.

In the field, water temperature, pH, electrical conductivity, total dissolved solids and dissolved oxygen were measured using a portable multi-parameter meter (Hengkaituo, Guangdong, China).

### Sample Preparation and Laboratory Analysis

Fresh carp samples (25 g consisting of about 20–30 individual carps) were homogenized in a stomacher bag containing 225 ml of alkaline peptone water, pH: 8.5 (APW) (Oxoid Ltd., Hampshire, United Kingdom). The homogenized samples contained carps with all body parts (skin, gills, intestines, flesh etc.). Hundred mL of water samples were filtered through a 0.45 μm pore diameter membrane (Millipore, Bedford, MA, United States) which was directly transferred into 225 mL APW; then the bottle was shaken vigorously before incubation. Similarly, phytoplankton was concentrated on a 0.45 μm pore diameter membrane filter paper (Millipore, Bedford, MA, United States) which was transferred into 225 mL APW. Samples in APW were enriched at 37°C for 6 h. Each sample was then analyzed as described below by a culture-based procedure and by PCR of DNA extracted from enriched APW samples.

After enrichment in APW, samples were streaked onto thiosulfate-citrate-bile salts-sucrose agar (TCBS) plates (Oxoid Ltd) and incubated at 37°C for 18–24 h. Characteristic dark yellow colonies with about 2 mm diameter were selected, purified and subsequently characterized by biochemical tests for identification of *V. cholerae* (Hounmanou et al., 2016). Pure cultures of presumptive *V. cholerae* colonies were then subjected to DNA extraction. DNA was extracted from boiled lysates of isolates and subjected to PCR for detection of the outer membrane protein gene (*omp*W) generating a 588 bp amplicon confirming them as *V. cholerae* (Dalusi et al., 2015a). Isolates confirmed as *V. cholerae* were subjected to agglutination with polyvalent *V. cholerae* O1 antiserum (Bio-Rad, France) and were tested for toxigenicity by PCR targeting the cholera enterotoxin subunit A gene (*ctx*A) along with the *omp*W primers in a multiplex reaction.

### Antimicrobial Susceptibility Testing

Strains confirmed as toxigenic *V. cholerae* O1 were subjected to antimicrobial susceptibility testing by the Kirby-Bauer disk diffusion method with *Escherichia coli* ATCC 22925 included for quality control. Twelve different antimicrobial disks (Oxoid Ltd) were used and results interpreted according to the Clinical and Laboratory Standards Institute (CLSI) guidelines (CLSI, 2017). Antimicrobials tested included: streptomycin (10 μg), gentamicin (30 μg), ampicillin (10 μg), nalidixic acid (30 μg), ciprofloxacin (5 μg), trimethoprim (5 μg), ceftazidime (30 μg), tetracycline (30 μg), chloramphenicol (30 μg), amoxicillin-clavulanicc acid (30 μg), cefotaxime (30 μg), and sulfamethoxazole (25 μg).

### Detection of Toxigenic *V. cholerae* by PCR From Enriched Samples

About 10 ml aliquots of APW enriched samples were stored at -20°C until analysis. Total DNA was extracted using a Zymo DNA extraction Kit (The Epigenetics Company, CA, United States) following the manufacturer’s protocol. DNA was extracted from 200 randomly selected samples of the 360 samples enriched in APW. A multiplex PCR was performed to target the 588-bp region of the *omp*W gene (*V. cholerae* species specific) and a 301-bp region of the subunit A of the cholera enterotoxin gene (*ctx*A). Samples that generated only one band (588 bp) were *V. cholerae* whereas samples showing both bands (588 and 301 bp) were concluded toxigenic *V. cholerae ctx*A-positive.

Primers used and PCR conditions were as previously described (Nandi et al., 2000; Dalusi et al., 2015a) with a modification of the annealing temperature which was set at 55.6°C during optimization.

### Plankton Diversity

Immediately after sampling in dark bottles, 250 mL phytoplankton samples were fixed with five mL Lugol’s solution and 10% formalin and then transported to the Water Quality Laboratory of the Lake Zone in Mwanza. Using an inverted microscope at resolutions of 40 × 10 and 10 × 10 and following standards keys (Verlecar and Desai, 2004), two ml of fixed sample was used by a trained taxonomist to identify taxons and species groups of phytoplankton present in the water samples.

### Statistical Analysis

Proportions of positive *V. cholerae* samples from the culture-based method and PCR of DNA extracted from APW enriched samples were compared using chi-square in Epi-Info software version 7.2^1^ at a probability of 0.05. Seasonal differences were also tested. Using the statistical software R v3.5.1^2^ logistic regression was performed to estimate the effect of water temperature, pH, conductivity, dissolved oxygen, and total dissolved solids on the occurrence of *ctx*A-positive *V. cholerae* between sampling points.

### DNA Extraction and Whole Genome Sequencing (WGS)

Eight of the ten *ctx*A-positive *V. cholerae* O1 recovered from fish, phytoplankton and water and two *ctx*A-positive non-O1 *V. cholerae* from phytoplankton and fish (Plankton1, Water1) were selected for WGS analysis. DNA was extracted using the automated Maxwell DNA extraction system (Promega Maxwell RSC, Madison, WI, United States) and the Maxwell DNA extraction and purification kit. Prior to extraction, samples were treated with Proteinase K (Sigma-Aldrich, St. Louis, MO, United States) at 56°C for 1 h followed by 10 min RNase (Sigma-Aldrich) treatment at room temperature. Concentrations of the extracted DNA were determined using a Qubit dsDNA HS assay kit (Invitrogen, United States). The DNA was run in 1% agarose gel to check for quality followed by the preparation for Illumina paired-end WGS using Illumina Miseq (Illumina, Inc., San Diego, CA, United States) according to the procedures previously described (Kaas et al., 2016). Raw sequences are submitted to the European Nucleotide Archive under the project number PRJEB30604 with the accession numbers of each sample indicated in Supplementary Table S1.

### *In silico* Serogroup Typing, Multi-Locus Sequence Typing, Determination of Major Virulence Genes, Pathogenicity Islands and Phage Susceptibility

The raw paired-end reads were assembled using SPAdes assembler (Bankevich et al., 2012) available online^3^. Assembled sequences were analyzed in the batch upload pipeline of the CGE platform^4^as previously described (Thomsen et al., 2016), where KmerFinder 2.1 identified the species and possible contaminations. The species *V. cholerae* was further confirmed based on the species-specific *omp*W gene using MyDbFinder 1.2 tool (Siriphap et al., 2017) with a threshold set at 98% identity. This revealed the closest genome to the analyzed sequences as *V. cholerae* 2010EL-1786 (Reimer et al., 2011). Moreover, the sequences were further analyzed using MyDbFinder 1.2 tool with default options to identify known virulence genes and pathogenicity islands (Supplementary Table S1). This included the identification of *V. cholerae* serogroup-specific genes (*rfbV*-O1, *wbfZ*-O139), biotypes-specific genes (*ctx*B, *rst*R, *tcp*A), putative virulence genes (including *ctx*A), and *VC2346* specific for the 7th pandemic *V. cholerae* using a threshold of 98% identity (Kaas et al., 2016; Siriphap et al., 2017). MyDbFinder coupled with nucleotides BLAST were used to genotype the strains based on the *ctx*B of the CTX prophage that they carried. This helps to identify the wave of the seventh pandemic that the strains belonged to using *ctx*B1, *ctx*B3, and *ctx*B7 sequences (Naha et al., 2012; Kaas et al., 2016; Rashid et al., 2016). Detection of genomic islands of *V*. *cholerae* mainly VPI-1, VPI-2, VSP-1, VSP-2, was also carried out *in silico*. Moreover, *mak*A, *als*A and Type VI secretion system (T6SS) genes were searched. The CTX prophage genomic region containing the core region and RS1 was also assessed in the samples. Detection of the PICI like elements, i.e., PLE1 and PLE2, responsible for phage susceptibility in *V. cholerae* was performed using MyDbFinder 1.2. The MLST 2.0 tool reached through the batch upload pipeline analyzed the sequence types (ST) of the *V. cholerae* strains in order to establish genetic relatedness with known STs that have been implicated in cholera outbreak. The analysis was based on the seven housekeeping genes: *adk, gyrB, metE, mdh, pntA, purM*, and *pyrC* (Kaas et al., 2016). Moreover, a ribosomal multi-locus sequence typing which is a more discriminative MLST typing method based on variations of the 53 genes encoding the bacterial ribosome protein subunits (*rps* genes) was used to investigate differences among the environmental strains as previously described (Jolley et al., 2012).

### Identification of Antimicrobial Resistance Genes, SXT Element, Class 1 Integron and Plasmids Typing

In the batch upload pipeline (Thomsen et al., 2016), ResFinder 3.0 assessed acquired antimicrobial resistance (AMR) genes in the assembled sequences. In ResFinder with default options, we also searched for beta-lactam resistance genes including *blaVCC-1*, a carbapenamase gene that is emerging in environmental *V. cholerae* as recently reported in strains from Canada and Germany (Mangat et al., 2016; Hammerl et al., 2017).

Detection of AMR genotype was further strengthened with the search for mobile genetic elements using MyDbFinder 1.2 (Larsen et al., 2012) for the detection of the SXT integrative conjugative element, the different classes of integrons, and the presence of mutations in the DNA gyrase (*gyr*A gene) and in the DNA topoisomerase IV (*par*C gene) (Siriphap et al., 2017). MyDbFinder 1.2 was used to compare sample sequences with the integrating conjugative elements (ICE) of *V*. *cholerae* O1 ICE*Vch*Hai1(JN648379) and of SXTMO10 (AY034138) at a threshold of 98% identity. Due to high levels of mutations in the SXT element (Wang et al., 2016) the threshold for detection of *int*SXT was set at 95% for % ID of 40% minimum length.

The search for plasmids was done in three steps. The PlasmidFinder 1.3 tool of the batch upload pipeline in CGE (Thomsen et al., 2016) was used to search for plasmid replicons. Due to the limitation of PlasmidFinder, which detects only replicons that are available in the CGE database, we conducted another specific search. This second step used MyDbFinder 1.2, where a local search was performed between our samples’ genomes and sequences of representative IncA/C plasmids (known for carrying multidrug resistance genes in *V. cholerae*) available from the Genbank (Accession numbers: KY399978, KM083064, KF551948, CP007636, CP033514, and KJ817377) (Carraro et al., 2014; Folster et al., 2014; Wang et al., 2018). This step also involved two cryptic plasmids recently isolated in *V. cholerae* strains (Acc. KY486774 and KY486775) (Ceccarelli et al., 2017). In the third step, we used Blast atlas in GView^5^ where genomes were analyzed against reference plasmids.

Moreover, the genome sequences were annotated in RAST v.2.0 (Brettin et al., 2015) and each annotated file was analyzed through the SEED viewer (Overbeek et al., 2014) for subsystem categorization of the genetic elements involved in survival and persistence in the aquatic environment.

### Single Nucleotide Polymorphism-Based Phylogenetic Analyses

To further identify evolution and genetic similarities within and between the environmental *V. cholerae* isolates as compared to cholera outbreak strains, assembled genomes were analyzed using CSIPhylogeny version 1.4 with default options (Kaas et al., 2014), where high quality SNPs of the environmental genomes were identified. Sequences of *V. cholerae* from Uganda and Kenya that have borders to Lake Victoria as well as previous outbreak strains from Tanzania were obtained from the GenBank and compared with our environmental strains to determine genome wide SNPs. Additionally, *V. cholerae* O1 outbreak strains from other countries of the African Great Lakes region including the Democratic Republic of Congo (DRC), Burundi and Rwanda but also from Zambia were included in the tree to access the spatial-temporal phylogenetic evolution of the environmental strains. Accession numbers of strains used in the SNP tree are reported in Supplementary Table S2. This generated a regional and time-scale phylogenetic tree showing the genetic relatedness and evolution between the environmental non-outbreak *V. cholerae* O1 strains and pandemic strains from the African Great Lakes region. The reference genome of *V. cholerae* O1 strain N16961 (Biosample SAMN02603969) was used to root the tree (Kaas et al., 2016). The Newick files obtained in CSIPhilogeny 1.4 were downloaded and the final tree was amended in iTOL^6^.

## Results

### Detection of *V. cholerae* in Samples From Lake Victoria

Using standard bacteriological procedures followed by PCR for the species-specific gene *omp*W, 108 isolates were identified as *V. cholerae.* These strains were isolated from 77 out of 360 samples (21.4%) analyzed with up to three characteristic colonies being selected from each TCBS agar plate for confirmation. However, APW-enriched samples subjected directly to multiplex PCR yielded 138 DNA samples that were positive for *V. cholerae* (*omp*W) out of the 200 samples selected for analysis (69.0%). A Chi-square comparison revealed that PCR of DNA extracted directly after enrichment in APW recovered significantly higher proportions of *V. cholerae*-positive samples than identification of *V. cholerae* from isolates obtained on TCBS agar plates (69% vs. 21.4%, *p* < 0.05). A total of 22 *V. cholerae* isolates out of the 108 isolates originally recovered were positive for the subunit A of the cholera enterotoxin gene (*ctx*A). Ten of these strains belonged to serogroup O1 and originated from phytoplankton (five isolates), carps (three isolates), and water (two isolates) samples. Phytoplankton collected offshore by the vertical sampling method yielded all five *V. cholerae* O1 strains from this source. Two water samples collected offshore contained *V. cholerae* O1 (Table 1).

**Table 1:** Detection of toxigenic *V. cholerae* O1 by culture and PCR.

Sample type	Sampling	Number of samples	Total *ctx*A-positive samples^a^	Total *ctx*A-positive O1 samples^a^	Total *ctx*A positive samples (APW-PCR)^b^
Phytoplankton	Horizontal method	60	4	0	5
	Vertical method	60	6	5	9
Water	Onshore	60	4	0	4
	Offshore	60	3	2	2
Carps	Landing sites	120	5	3	3

*^*a*^Strains isolated on TCBS agar and confirmed by PCR; ^*b*^Toxigenic *V. cholerae* identified by PCR of samples pre-enriched in alkaline peptone water (APW).*

In DNA extracted from APW enriched samples, 23 samples contained the *ctx*A gene in phytoplankton (14), water (6) and carps (3). Fifteen of the PCR-positive samples did not yield any colonies by the culture-based technique. The vertical phytoplankton sampling method yielded slightly more toxigenic *V. cholerae ctx*A-positive samples than the horizontal sampling method (*p* > 0.05), which was also the case for samples collected onshore compared with offshore water samples (Table 1). The total DNA extracted from samples enriched in APW were however, not used for further characterization because the quality of the DNA does now allow for sequencing analysis.

### Seasonal Variation of *V. cholerae* in Lake Victoria

There was no statistical seasonal difference (*p* > 0.05) in recovery of *V. cholerae* when DNA was obtained from pure cultures (32.8 vs. 27.2%, dry and rainy seasons, respectively) compared to when DNA was obtained from APW-enriched samples (71.0 vs. 67.0%). Nevertheless, a statistically higher proportion of *ctx*A-positive *V. cholerae* was obtained in the dry season as compared to the rainy season for samples analyzed by culture procedures (*p* = 0.04).

### Physico-Chemical Water Parameters and Phytoplankton Diversity

The water temperature at the different sampling sites ranged from 24.9 to 25.9°C and water pH ranged between 7.5 and 8.5. The electrical conductivity fluctuated between 90 and 150 μS/cm (Supplementary Table S3). None of these parameters varied significantly between sampling points when they were regressed on the total toxigenic *V. cholerae ctx*A-positive samples (*p* > 0.05). The analyzed phytoplankton samples contained 45 different taxa belonging to three major phytoplankton groups including green algae (62.2%), cyanobacteria (31.1%), and diatoms (6.7%).

### Genomic Characterization of the *V. cholerae* Strains

We sequenced ten strains including eight of the isolated *ctx*A-positive *V. cholerae* O1 and two *ctx*A-positive *V. cholerae* non-O1, which were characterized by analysis of WGS data. Based on WGS analysis, all the ten sequenced strains including the two phenotypically non-O1 contained the *rfbV-O1* gene and should therefore be regarded as of serogroup O1. Table 2 shows genomic characteristics of the strains when variations was seen (see further details in the Supplementary Table S1), whereas similar characteristics are described in the text below. Most strains belong to the third wave of the seventh pandemic as they are all atypical El Tor biotype variants of *V. cholerae* O1, carrying the *ctx*B7 genotype of the *ctx*B gene while possessing the *rst*R and *tcp*A genes of El Tor biotype. However, strains Water2, Fish1, Fish3 possess *ctx*B1 of the early third wave and Plankton1 contained *ctx*B3 of the first wave clustering with older outbreak strains. The *in silico* MLST revealed that all strains belonged to the same sequence type ST69 and harbored the seventh pandemic-specific gene (VC2346) suggesting that they belong to the same clonal linage.

**Table 2:** Genomic sequence data, virulence profile and occurrence of antimicrobial resistance genes in the *V. cholerae* strains.

Strain ID	No. of contigs	Genome size (bp)	rMLST	intSXT	SXT/R391
Fish1	63	40505002	rST14417	+	ICEVchHai1
Fish2	71	4029796	rST78290	+	ICEVchHai1_del^a^(floR,strA/B,sul2)
Fish3	63	40505002	rST14417	+	ICEVchHai1
Fish4	71	4029706	rST78290	+	ICEVchHai1_del(floR,strA/B,sul2)
Plankton1	127	4009065	rST78277	–	Not Found
Plankton2	81	4022914	rST78290	+	ICEVchHai1_del(floR,strA/B,sul2)
Plankton3	89	4008427	rST78290	+	ICEVchHai1_del(floR,strA/B,sul2)
Water1	84	4022791	rST78290	+	ICEVchHai1_del(floR,strA/B,sul2)
Water2	66	4048306	rST14417	+	ICEVchHai1
Water3	98	4030711	rST78290	+	ICEVchHai1_del(floR,strA/B,sul2)

*^*a*^del, deletions in specified genes.*

The occurrence of virulence-associated genes and pathogenicity islands among the environmental *V*. *cholerae* was similar in all sequenced strains. This included the major virulence-associated genes such as *ctxA, ctxB, zot, ace, tcpA, hlyA, mshA, rtxA, ompU*, and *toxR*, whereas *stn* and *chxA* genes were absent. Moreover, the strains all contained genes of the Type VI secretion system, the gene involved in glucose metabolism, *als* and the flagella-mediated cytotoxin gene *mak*A. All strains contained the pathogenicity islands VPI-1, VPI-2, VSP-1, and VSP-2.

### Phenotypic and Genotypic Antimicrobial Resistance, Mobile Genetic Elements and Phage Resistance

**FIGURE 2:**
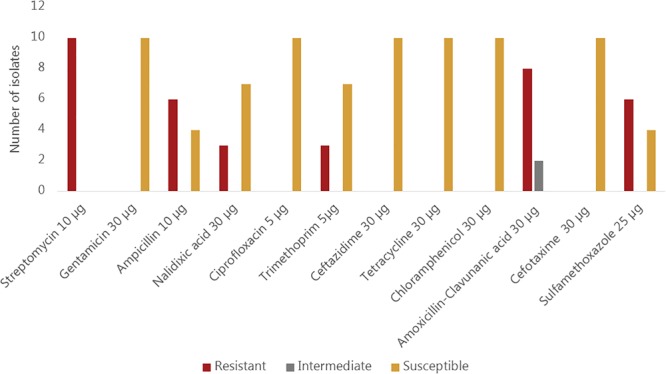
Phenotypic antimicrobial susceptibility of environmental *Vibrio cholerae* O1 strains.

All ten sequenced *V. cholerae* strains showed phenotypic resistance to streptomycin, while eight of them showed resistance to amoxicillin and clavulanic acid. Phenotypic resistance to ampicillin and sulfamethoxazole was observed in 6/10 strains whereas resistance to nalidixic acid and trimethoprim was observed in three strains. All isolates were susceptible to gentamicin, ciprofloxacin, ceftazidime, tetracycline, chloramphenicol and cefotaxime (Figure 2). Sequence analysis revealed, however, that some genes encoding resistance to certain antimicrobial classes were present in strains that are phenotypically susceptible. For instance, all strains harbored the *cat*B9 gene conferring resistance to chloramphenicol, but were phenotypically susceptible. Similarly, all strains except the strain Plankton1 harbored the *dfr*A1 gene conferring resistance to trimethoprim, while only three of them showed phenotypic resistance.

**FIGURE 3:**
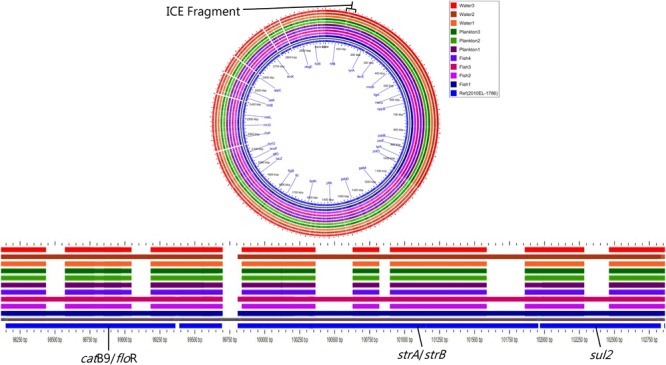
Nucleotide deletions in resistance genes on the ICE fragment.

Moreover, all strains except Plankton1 contained the SXT integrative conjugative element (*int*SXT gene), but they all lacked specific integrase genes of the class 1 integron (*int*I gene). Nevertheless, not all the antimicrobial resistance genes normally found on the SXT element such as *sul*2, *dfr*A1, *dfr*A18, *flo*R, *str*A, and *str*B which are associated with sulfamethoxazole and trimethoprim, chloramphenicol and streptomycin resistance, respectively were present in the strains according to MyDbFinder 1.2. Therefore, a Blast Atlas analysis was performed where the genomes where compared to the reference *V. cholerae* strain 2010EL-1786. This revealed that except for strains Fish1, Fish3, and Water2 that were positive for aminoglycoside, phenicol and sulfonamide resistance genes, the remaining strains showed nucleotide deletions in their genomes (Figure 3). A more detailed analysis of the strains that have deletions showed that they contained fragments of the concerned genes (*str*A, *str*B, *Sul*2, *flo*R) on the ICE fragment, with about 1100 bp gaps between bp position 98500–102450 (Figure 3).

Strains Fish1, Fish3, and Water2 showed resistance to nalidixic acid and contained expected amino acid substitutions in gyrA (Ser83-Ile) and parC (Ser85Leu), but none of the ten strains contained the fluoroquinolone resistance gene *qnr*VC1. Likewise, none of the ten strains contained beta-lactam resistance genes including *blaVCC-1*, a carbapenamase gene. Nine environmental strains harboring the SXT element had a genomic organization of the integrating conjugative element (ICE) similar to that of the *V. cholerae* ICEVchHai1 (Reimer et al., 2011) reference strain with some common deletions seen in loci VC1786ICE78 for all strains and in VC1786ICE14 for strains Fish1, Fish3 and Water2. The other six strains had deletions in loci VC1786ICE6-13, VC1786ICE81, and VC1786ICE83-84 (Supplementary Table S4).

Our strains have no plasmid replicons according to PlasmidFinder. Moreover, in MyDbFinder and in Blast atlas, the strains had no copies of the IncA/C plasmid, as well as the cryptic plasmids pSDH1-2. The 10 strains irrespective of their biotype lacked the phage susceptibility region of the PICI like elements (PLE1, PLE2).

### Genetic Elements Supporting Environmental Persistence in Studied Genomes

**FIGURE 4:**
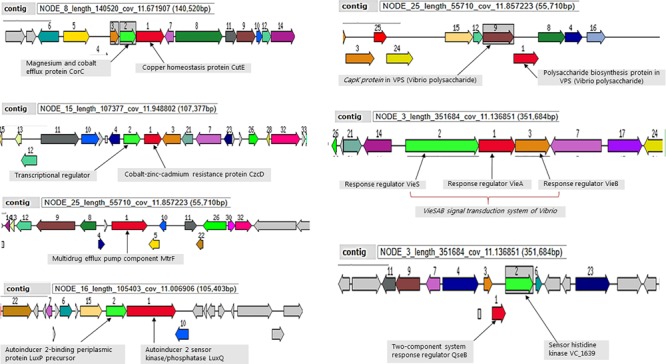
Selected genetic elements encoding for environmental fitness. Strain Plankton2 was used for this illustration. The contig in which the genetic complex are found is indicated.

All ten analyzed genomes present molecular machinery for attachment, survival and defense for environmental persistence. The magnesium and cobalt efflux protein (*Cor*C), the cobalt-zinc-cadmium resistance protein (*Czc*D) and the multidrug efflux pump component (*Mtr*F) were conversed in the defense system of all the sequenced strains (Figure 4). Moreover, the strains all possess the *msh*A gene involved in attachment to chitin, as well as autoinducers (AI-2 LuxP and LuxQ) involved in quorum sensing and biofilm formation for environmental survival. A number of environmental stress response regulator proteins were conserved in the strains mainly the response regulators of the *Vie*SAB transduction system of *Vibrio*, the two-component response regulator proteins, histidine kinase, and Vibrio polysaccharides (VPS) biosynthesis proteins (Figure 4).

### Phylogenetic Comparison Analysis of the Environmental *V. cholerae* O1 and Cholera Outbreak Strains in Countries Surrounding Lake Victoria

Overall, the SNP tree revealed a wide diversity among the environmental strains with up to 174 SNP differences (found between strains Plankton1 and Plankton3). Strains Fish1 and Fish3, as well as Fish2 and Fish4 were strictly clonal, respectively. Very close relatedness was also observed between strains Water2, Fish1, and Fish3 (only four SNPs difference), between Water2, Fish2, and Fish4 (only five SNPs difference) and between strains Plankton2, Fish2, and Fish4 (only nine SNPs difference). We further performed a ribosomal MLST typing (Jolley et al., 2012) to assess the diversity of the environmental strains and found little diversity as six of the ten strains belong to the same rMLST type ST78290. The strain Plankton1, which shows above 100 SNP differences compared to its pairs, belongs to a separate rMLST type 78277 whereas strains Fish1, Fish3, and Water2 belong to rMLST type 14417.

**FIGURE 5:**
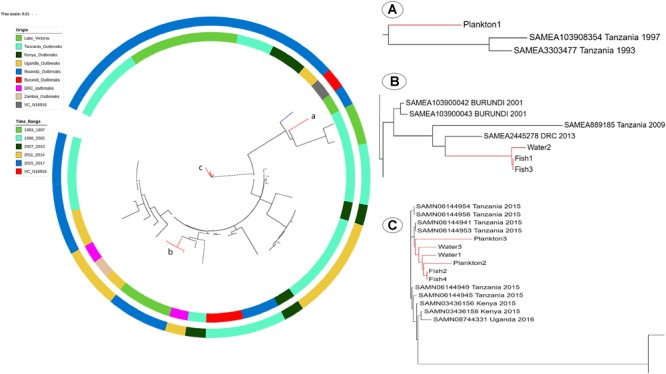
SNP-Tree showing spatial-temporal phylogeny of environmental strains and pandemic *V. cholerae* from seven countries in the African Great Lakes Region. Colors on the inner lane indicate the origin of strains and the outer lane colors indicate the timeline evolution of the strains as shown in the legend. The tree was rooted with the reference *V. cholerae* N16916 (blue branch). Red branches identify the position of environmental genomes on the tree. **(A)** Localization of the strain Plankton1 in a clade of older strains involved in the initial waves of the seventh pandemic; **(B)** position of Fish1, 3 and Water2, and **(C)** a clade of recent outbreak strains in Tanzania, Kenya, and Uganda with most environmental strains.

When compared to published genomes of the Genbank, the environmental *V. cholerae* O1 strains were found in three different clusters among the pandemic strains. The strain Plankton1 was distant from all genomes as a singleton but closer to strains associated with cholera outbreaks in Tanzania from 1993 and 1997 with 88 to 95 SNPs (Figure 5A and Supplementary Table S5). Strains Fish1, Fish3, and Water2, however, formed a separate cluster (29–33 SNPs, Supplementary Table S6) with a 2013 pandemic strain from DRC (Figure 5B). This clade also contains older pandemic strains from Burundi (2001) and Tanzania (2009). The other environmental strains were highly related to recent epidemic strains with less than 22 SNPs difference between our environmental O1 strains Fish2, Fish4, Plankton2, Plankton3, Water1, Water 3 and the *V. cholerae* O1 strains associated with cholera outbreaks in Tanzania and Kenya in 2015 and in Uganda in 2016 (Figure 5C). The strains Fish2 and Fish4 for instance, were highly clonal when compared to the 2015 Tanzanian and Kenyan outbreak strains (only three SNPs apart; Figure 5C) and the Uganda outbreak strain (nine SNP differences) (Biosample Accession SAMN08744331). The largest difference in SNPs between the environmental and pandemic *V. cholerae* O1 strains was 183, found between strain Plankton1 and one strain associated with the Ugandan cholera outbreak (Biosample Accession: SAMN08744333). The pairwise SNP differences among all strains are reported in Supplementary Table S5.

## Discussion

We report the occurrence of toxigenic *V. cholerae* O1 and non-O1 in the aquatic environment of the Tanzanian basin of Lake Victoria during a non-cholera outbreak period. The presence of cholera toxin-positive *V. cholerae* non-O1 in Lake Victoria suggests that the *ctx*A gene is not limited to only the O1/O139 serogroups. It should be noted that we did not agglutinate the non-O1 strains in O139 antiserum. However, *V. cholerae* O139 do not seem to occur in Africa and a recent report on non-toxigenic O139 needs to be confirmed (Bwire et al., 2018a). Studies have identified *V. cholerae* O141 and O75 harboring the CTX prophage including the *ctx*A gene which have been responsible for cholera-like outbreaks, e.g., in the United States (Dalsgaard et al., 2001; Crump et al., 2003; Haley et al., 2014). Moreover, the occurrence of non-O1 toxigenic strains in Lake Victoria underlines that serotyping beyond O1 and O139 antisera should be implemented as the CTX prophage seem to be present in more serogroups than initially documented (Waldor and Mekalanos, 1996).

The two toxigenic non-O1 *V. cholerae* sequenced in this study showed that they were in fact O1 serogroup strains based on the presence of the *rfb*V-O1 gene. This discrepancy between the phenotype and the genotype could be due to changes in the O antigen expression leading to delayed and non-conclusive agglutination in these strains during testing. It is unlikely that our phenotypically non-O1 *ctx*A-positive strains might be of another serogroup than O1 since they possess the *rfb*V-O1 gene but also belong to the atypical El Tor biotype of serogroup O1.

The most recent cholera outbreak in the study area occurred in Mwanza in March 2016 (Unpublished report from the Tanzanian Ministry of Health and Social Welfare). Thus, the current study was conducted more than a year after the latest reported cholera outbreak; yet toxigenic *V. cholerae* O1 strains were recovered from phytoplankton, fish (carps) and water samples, suggesting that Lake Victoria is a reservoir of *V. cholerae*. Isolation of *V. cholerae* O1 strains between inter-epidemic periods are rare (Alam et al., 2006; Faruque and Mekalanos, 2012) partly because *V. cholerae* O1 may become dormant, i.e., viable-but-non-culturable (Kamruzzaman et al., 2010; Bari et al., 2013). Nevertheless, our results are consistent with other findings, which also detected toxigenic strains of *V. cholerae* O1 in aquatic environments during non-cholera outbreak periods (Onyuka et al., 2011; Dalusi et al., 2015a; Hounmanou et al., 2016). Furthermore, the sequenced environmental *V. cholerae* isolates belong to the seventh cholera pandemic lineage similar to strains that caused recent epidemics (2015–2016) in countries surrounding Lake Victoria (Tanzania, Kenya, and Uganda) with SNPs ranging between 3 and 22 for samples Fish2, Fish4, Plankton2, Plankton3, Water1, and Water 3. This very close genetic relatedness to outbreak strains further substantiates that such environmental isolates are of outbreak potential given that their closest progenitors are epidemic strains. The environmental *V. cholerae* O1 isolates are likely of outbreak origin and potential progenitors of cholera outbreak strains. On the other hand, the spatial-temporal analysis of our environmental strains revealed a regional spread and long-term environmental persistence of pandemic *V. cholerae* within the African Great Lakes region. The close genetic relatedness of strain Plankton1 to the 1993 and 1997 pandemic strains from Tanzania suggests that this strain did not emerge from the recent outbreaks like the others but has probably persisted in the lake for at least 20 years. Although Plankton1 is an atypical El Tor strain whereas the 1993 and 1997 pandemic strains were prototype El Tor, their closer phylogenetic relatedness compared to the other environmental and pandemic strains show that this strain most probably underwent *ctx*B-mediated mutations throughout the years to become atypical El Tor (Rashid et al., 2016). Furthermore, Fish1, Fish3, and Water2 were distant from their environmental counterparts but closely related to older pandemic strains from DRC, Tanzania and Burundi. The finding of strains Fish1, Fish3, and Water2 suggests not only a long-term persistence of *V. cholerae* in the lake but also an environmental adaptation aided by a human-mediated spread between the countries in the African Great Lakes region enhanced by connecting water bodies. This phylogenetic heterogeneity of *V. cholerae* isolated in Lake Victoria substantiate that the lake serves as reservoir for long-term persistence of pandemic clones of *V. cholerae* with a much-dispersed geographical distribution. Sequence analysis revealed that the *V. cholerae* O1 strains possessed all major virulence-associated genes found in clinical O1 strains, e.g., the cholera enterotoxin genes, toxin co-regulated pilus, hemolysis genes, as well as the flagella-mediated toxin gene *mak*A (Aliabad et al., 2012; Castillo et al., 2018; Dongre et al., 2018). Genes involved in the T6SS that enables *V. cholerae* to overcome commensals and immune cells in the human gut during infection were also present in our environmental isolates (Unterweger et al., 2014; Logan et al., 2018). Moreover, the environmental strains were *V. cholerae* O1 El Tor variants of the Haitian *ctx*B7 genotype and therefore similar to strains implicated in the ongoing third wave of the seventh pandemic known to cause more severe disease as compared to the typical El Tor biotype (Ghosh-Banerjee et al., 2010; Mutreja et al., 2011; Kim et al., 2014). The El Tor biotype variants of *V. cholerae* O1 are proposed to be common in the African Great Lakes region with potential to cause cholera outbreaks (Echenberg, 2011). Our study provides evidence that cholera outbreak isolates may persist in the aquatic environment during non-outbreak periods. This may explain why clonal isolates are found to cause different outbreaks many years apart, e.g., as seen in Mozambique (Garrine et al., 2017).

*Vibrio cholerae* of sequence type ST69 belonging to the third wave of the seventh cholera pandemic are the ones responsible for most cholera outbreaks worldwide (Mutreja et al., 2011; Kachwamba et al., 2017; Weill et al., 2017). The presences of the pathogenicity islands VPI-1, VPI-2, VSP-1, and VSP-2 in the strains further confirm their outbreak potential. Our strains clustered in two major ribosomal MLST types mainly rMLST 78290 and 14417, which are identical to rMLST types of cholera outbreaks strains in the region (Mutreja et al., 2011; Kachwamba et al., 2017). We observed that strains of rMLST type 78920 clustered together on the SNP tree with outbreak strains of the same rMLST with strains of rMLST type 14417 clustering in a similar manner (Figure 5). Most cholera outbreak strains isolated from 2015 and the majority of our environmental strains are of an identical rMLST type 78920 suggesting not only a transmission of strains between the aquatic environment and humans, but also a time-scale evolution of *V. cholerae* from one rMLST type to another. Our results suggests that Lake Victoria is a reservoir for *V. cholerae* O1 with outbreak potential which are consistent with findings in previous studies (Faruque et al., 1998, 2007; Dalusi et al., 2015b), but are not in agreement with a recent report which questioned whether environmental strains may be progenitors of outbreak strains (Weill et al., 2017). In Haiti, a close phylogenetic relationship has also been reported between clinical and environmental toxigenic *V. cholerae* O1 strains (Azarian et al., 2014). Moreover, some *ctx*-negative environmental *V. cholerae* O1 clustered with strains responsible for clinical cholera and possessed genomic characteristics of the seventh pandemic lineages (Azarian et al., 2016). This provides further arguments that the aquatic environment is a reservoir in the survival and generation of progenitors of outbreak strains of *V. cholerae*.

The persistence of epidemic *V. cholerae* O1 strains in the lake can be attributed to a number of environmental factors. One is the presence of suitable hosts. The symbiotic relationship between phytoplankton and *V. cholerae* is well documented and known to enhance the survival and maintenance of the pathogenicity in these strains even after cholera outbreaks have ceased (Haque et al., 2012; Mitchell et al., 2017). Attachment to aquatic organisms to take advantage of chitin and other nutrients is dependent on the MSHA (mannose*-*sensitive hemagglutinin) pili (Meibom et al., 2004; Sinha-Ray and Ali, 2017), which were present in our *V. cholerae* strains and whose expression by the *msh*A gene increases in warm tropical water with temperatures above 15°C (Lutz et al., 2013). The conductivity measurements and the surface water temperature in the lake averaging 25°C with an alkaline pH averaging 8.5, provides optimum conditions for survival and growth of *V. cholerae* (Lugomela et al., 2014; Dalusi et al., 2015a; Plisnier et al., 2015). The WGS data analysis further indicates that the *V. cholerae* strains have survived in and adapted to the lake environment for about two decades. Among phytoplankton groups that may harbor *V. cholerae* O1 in Lake Victoria, green algae and cyanobacteria were the major species detected in our study which are both documented aquatic reservoirs for *V. cholerae* O1 and O139 in Asia and Africa (Eiler et al., 2007; Dalusi et al., 2015b; Islam et al., 2015). We noticed that most *V. cholerae* O1 strains were recovered in phytoplankton samples obtained in deep water which suggests that the vertical sampling technique for phytoplankton should be used in future environmental surveillance studies. Food safety aspects and transmission of *V. cholerae* O1 in carps to humans are questionable as carps are sun-dried before consumption, but needs to be further explored. *V. cholerae* O1 was isolated in Nile perch from the Kenyan side of the Lake Victoria (Onyuka et al., 2011), but the role of the small carps as reservoir hosts of *V. cholerae* O1 remains uncertain. Experimental studies suggest that *V. cholerae* strains can use accessory toxins and structural components to survive in the gut of zebrafish over long periods (Runft et al., 2014; Mitchell et al., 2017). Moreover, Hilsa fish from Bangladesh and many other species were also reported aquatic hosts of *V. cholera*e (Halpern and Izhaki, 2017; Hossain et al., 2018a).

Although seasonal variation was not a significant predictor of recovery of toxigenic strains in this study, *V. cholerae* O1 are generally more frequently isolated during rainy seasons (Reyburn et al., 2011). Our strains were negative for the PICI-like elements responsible for phage susceptibility. These elements are genetic islands of *V. cholerae* that inhibit lytic phages and therefore serve as a resilience factor toward vibrio phages (Seed et al., 2013). It has been shown that seasonal fluctuations of cholera cases can be associated with predation of *V. cholerae* O1 by phages in the environment leading to a decrease in the epidemic curve (Faruque and Mekalanos, 2012). A long-term persistence of epidemic *V. cholerae* O1 clones in the lake can also be attributed to their adaptive genetic machinery made of defense genes against toxic compounds and heavy metals. The strains contained magnesium and cobalt efflux protein (*Cor*C) and the cobalt-zinc- cadmium resistance protein (*Czc*D). Studies have found different levels of heavy metals and pesticide residues in Lake Victoria (Kishe and Machiwa, 2003; Ogwok et al., 2009), which could be detrimental to bacteria like *V. cholerae* and limit their growth. The capability of *V. cholerae* O1 strains to survive in such an environment is justifiable by the genetic elements enhancing their ability to resist these toxic compounds. Moreover, in a complex environment like Lake Victoria, *V. cholerae* may be subjected to a number of other environmental stresses. The presence of autoinducers, AI-2 LuxP and LuxQ (Joelsson et al., 2007) in the genetic makeup of our strains favors their persistence since they are capable of quorum sensing and forming biofilm to survive and thrive (Kamruzzaman et al., 2010; Bari et al., 2013; Sinha-Ray and Ali, 2017). The strains also contained various stress response regulator proteins like the *Vie*SAB transduction system, the two-component response regulator proteins, and Vibrio polysaccharides (VPS) biosynthesis proteins, which are essential for environmental fitness and persistence in *V. cholerae* (Fong et al., 2010; Vesth et al., 2010; Lutz et al., 2013). Thus, the molecular characteristics of our *V. cholerae* strains substantiate their spatial-temporal persistence in the lake from where they may emerge and cause outbreaks.

As the concentration of *V. cholerae* O1 is low in the aquatic environment between cholera outbreaks, it is important to apply methods with high sensitivity for environmental surveillance (Bwire et al., 2018a; Nyambuli et al., 2018). We observed that PCR of DNA extracted from APW-enriched samples detected more *V. cholerae*-positive samples than using subculture onto TCBS agar with subsequent confirmation of selected isolates by PCR. Similar observations were reported analyzing sediments and plankton from Tanzanian estuaries using culture-independent PCR techniques (Dalusi et al., 2015a). Viable but non-culturable forms of toxigenic and non-toxigenic *V. cholerae* are important in the ecological dynamics and epidemiology of cholera (Kamruzzaman et al., 2010; Faruque and Mekalanos, 2012). However, these forms of *V. cholerae* cells cannot be detected by culture directly on selective agar media. We therefore suggest the detection of toxigenic *V. cholerae* by PCR of APW-enriched samples as a sensitive and efficient method in routine monitoring of *V. cholerae* in the environment.

Increasingly, antimicrobials are used in cholera treatment to reduce the volume of diarrhea, and *V. cholerae* O1 concentrations in stools. Our study revealed that isolates were resistant to commonly used antimicrobials such as streptomycin, amoxicillin + clavulanic acid, ampicillin, trimethoprim and sulfamethoxazole; findings which are similar to earlier studies of *V. cholerae* from Tanzania (Hounmanou et al., 2016; Rabia et al., 2017). Most of our strains carried the SXT integrative conjugative element containing resistance genes to aminoglycosides, sulfamethoxazole, trimethoprim, phenicols and quinolones with genomic similarity to *V. cholerae* ICEVchHai1 (Hendriksen et al., 2011; Kaas et al., 2016). Some of our strains carrying genes encoding phenicol resistance were susceptible to chloramphenicol (Siriphap et al., 2017; Hossain et al., 2018b) which shows that the expression of a phenotype is not solely related to the possession of encoding gene. Moreover, the nucleotide deletions that occurred within the SXT element are not rare, as studies have underlined frequent mutation rates in the ICE cassette (Spagnoletti et al., 2014; Wang et al., 2016; Hossain et al., 2018b). This could also explain why the strain Plankton1, which was negative for the SXT element, was genetically distinct from the rest (Figure 5). Plasmids were not a significant factor encoding antimicrobial resistance because antimicrobial resistance genes in our *V. cholerae* strains was rather related to the SXT element (Wang et al., 2016). The lack of plasmids in our strains may also explain the absence of beta-lactam genes as beta-lactamases are commonly carried by conjugative plasmids (Kudirkiene et al., 2018). Nevertheless, some cryptic plasmids have been found in non-O1 strains (Ceccarelli et al., 2017), while the IncA/C conjugative plasmids responsible for multidrug resistance were detected in some *V. cholerae* in Haiti (Wang et al., 2018). Acquisition of these resistance profiles not only is of clinical therapeutic relevance but may also be part of the defense mechanisms of *V. cholerae* in aquatic environments as protection against antimicrobial compounds and antimicrobial residues that may be present in the lake. Besides the ICE element, all sequenced strains contained a multidrug efflux pump component (*Mtr*F) which could support their ability to persist in the lake in case of exposure to antimicrobial residues favoring their environmental persistence as it may also enhance their interaction with aquatic organisms (Alvarez-Ortega et al., 2013), as well as virulence (Alcalde-Rico et al., 2016).

This study reports the occurrence of multidrug resistant *V. cholerae* O1 in Lake Victoria that are genetically closely related to recent pandemic strains in Tanzania, Kenya and Uganda. The strains identified are also closely related to older pandemic strains recovered in the Democratic Republic of Congo, Burundi and Tanzania up until 1993, suggesting a long-term persistence and wide spatial distribution of pandemic strains within the region with the lake serving as a reservoir. These environmental isolates likely emerged from previous cholera outbreaks and survived in the lake environment for decades through various relations with reservoirs such as phytoplankton and fish. The *V. cholerae* O1 strains in the lake are potential progenitors of future cholera outbreak strains. Our findings are important for surveillance of *V. cholerae* O1 and understanding the epidemiology of cholera in countries around the lake.

## Ethics Statement

The present study required no ethical approval since the analyzed samples were collected from the environment, namely water, phytoplankton and carp from Lake Victoria. We, however, obtained a research permit from local authorities in Mwanza, the study area for collection of these samples in the Mwanza basin of Lake Victoria. The permit issued in Kiswahili Language is submitted with the Manuscript only for editorial use.

## Author Contributions

YH collected samples and carried out the study in the laboratory, analyzed the results, and drafted the manuscript. PL participated in genomic data analysis, critical reviewing and editing of original draft manuscript. RH provided guidance and participated in genomic data analysis, critical reviewing, and editing of the manuscript. TD participated in critical reviewing and editing of the manuscript. RM, JO, and AD conceived, designed, and contributed to the revision of the draft and final approval of the version to be published. AD was the principal supervisor of the project. All authors read and approved the final manuscript.

## Conflict of Interest Statement

The authors declare that the research was conducted in the absence of any commercial or financial relationships that could be construed as a potential conflict of interest.

## Footnotes

1 https://www.cdc.gov/epiinfo/index.html

2 https://cran.r-project.org/

3 https://cge.cbs.dtu.dk/services/SPAdes/

4 https://cge.cbs.dtu.dk/services/cge/

5 https://server.gview.ca/

6 https://itol.embl.de/
