# Corrigendum: Surveillance and Genomics of Toxigenic *Vibrio cholerae* O1 From Fish, Phytoplankton and Water in Lake Victoria, Tanzania

**DOI:** 10.3389/fmicb.2019.02974

**Published:** 2019-12-20

**Authors:** Yaovi M. Gildas Hounmanou, Pimlapas Leekitcharoenphon, Rene S. Hendriksen, Tamegnon V. Dougnon, Robinson H. Mdegela, John E. Olsen, Anders Dalsgaard

**Affiliations:** ^1^Department of Veterinary and Animal Sciences, Faculty of Health and Medical Sciences, University of Copenhagen, Copenhagen, Denmark; ^2^National Food Institute, WHO Collaborating Center for Antimicrobial Resistance in Food Borne Pathogens and Genomics and European Union Reference Laboratory for Antimicrobial Resistance, Technical University of Denmark, Kongens Lyngby, Denmark; ^3^Research Unit in Applied Microbiology and Pharmacology of Natural Substances, Laboratory of Research in Applied Biology, Polytechnic School of Abomey-Calavi, University of Abomey-Calavi, Cotonou, Benin; ^4^Department of Veterinary Medicine and Public Health, College of Veterinary Medicine and Biomedical Sciences, Sokoine University of Agriculture, Morogoro, Tanzania

**Keywords:** *Vibrio cholerae*, genomics, aquatic reservoirs, African Great Lakes, microbial ecology

In the original article, there was a mistake in Supplementary Table S1 as published. **In row number 17, all *ctx*B genotypes were *ctx*B7**. The corrected Supplementary Table S1 contains ***ctx*B1** in columns D, F, and L and ***ctx*B3** in column H.

In the original article, there was an error. “All strains belong to the third wave of the seventh pandemic as they are all atypical El Tor biotype variants of *V. cholerae* O1, carrying the *ctx*B7 genotype of the *ctx*B gene while possessing the rstR and tcpA genes of El Tor biotype.”

A correction has been made to the ***Results Section***, ***Sub-section: “***Genomic Characterization of the *V. cholerae* Strains,” ***Paragraph Number 1***:

“Most strains belong to the third wave of the seventh pandemic as they are all atypical El Tor biotype variants of *V. cholerae* O1, carrying the *ctx*B7 genotype of the *ctx*B gene while possessing the rstR and tcpA genes of El Tor biotype. However, strains Water2, Fish1, Fish3 possess *ctx*B1 of the early third wave and Plankton1 contained *ctx*B3 of the first wave clustering with older outbreak strains.”

The authors apologize for this error and state that this does not change the scientific conclusions of the article in any way. The original article has been updated.

## Supplementary material

The Supplementary Material for this article can be found online at: https://www.frontiersin.org/articles/10.3389/fmicb.2019.02974/full#supplementary-material
