# Supplementary material for: Surveillance and Genomics of Toxigenic Vibrio cholerae O1 From Fish, Phytoplankton and Water in Lake Victoria, Tanzania
Source: Front Microbiol. 2019 Apr 30;10:901. doi: 10.3389/fmicb.2019.00901 (PMC6503148; doi:10.3389/fmicb.2019.00901)
Supplement: Supplementary file 3 [file Table_3.DOCX]

**Table S3.** Average Physico-chemical measures of water at sampling points

| **Sites** | **Total *ctx*A positive samples** | **Depth (m)** | **Dissolved Oxygen**  **(mg/L)** | **Temperature**  **(°C)** | **pH** | **Conductivity**  **(µS/cm)** | **Total Dissolved Solids**  **(mg/L)** | **GPS Coordinates** |
| --- | --- | --- | --- | --- | --- | --- | --- | --- |
| Mkuyuni | 5 | 6.2 | 6.3 | 24.9 | 7.7 | 100 | 50 | -2.536485S 32.897556E |
| Kijiweni | 4 | 8.1 | 8.2 | 25.7 | 8.5 | 90 | 45 | -2.584129S 32.886083E |
| Shadi | 4 | 5 | 6.3 | 25.6 | 8.2 | 100 | 45 | -2.646362S 32.872554E |
| Igombe | 3 | 13.1 | 6.8 | 25.5 | 7.5 | 120 | 40 | -2.398461S 32.952028E |
| Kayenze | 6 | 6 | 9.6 | 25.9 | 8 | 150 | 45 | -2.386833S 33.083197E |
| Mihama | 10 | 16.8 | 6.1 | 25.1 | 7.6 | 90 | 45 | -2.446679S 32. 876290E |
| Bwiru | 3 | 18.3 | 4 | 25 | 8 | 95 | 45 | -2.107903S 32. 903694E |
| p-value (Regression) |  | 0.7 | 0.74 | 0.7 | 0.47 | 0.8 | 0.6 |  |
| R-squared (%) |  | 3 | 2.4 | 2.5 | 10 | 0.4 | 5 |  |
